# Supplementary material for: Effectiveness of Heterologous and Homologous Ad26.COV2.S Vaccine Boosting in Preventing COVID-19-Related Outcomes Among Individuals with a Completed Primary Vaccination Series in the United States
Source: Vaccines (Basel). 2025 Feb 9;13(2):166. doi: 10.3390/vaccines13020166 (PMC11861575; doi:10.3390/vaccines13020166)
Supplement: Supplementary file 1 [file vaccines-13-00166-s001.zip › vaccines-3436017-supplementary.pdf]

Supplemental Materials

Supplemental Table S1. Variable Definitions

Variable name	Description
Ad26.COV2.S (Janssen)	Any medical claim, pharmacy claim, inpatient hospital encounter, or outpatient hospital encounter with one of the following CPT codes:<br>● 91303 - Severe acute respiratory syndrome coronavirus 2 (SARS-CoV-2) (coronavirus disease [COVID-19]) vaccine, DNA, spike protein, adenovirus type 26 (Ad26) vector, preservative free, 5x1010 viral particles/0.5mL dosage, for intramuscular use<br>● 0031A - Immunization administration by intramuscular injection of severe acute respiratory syndrome coronavirus 2 (SARS-CoV-2) (coronavirus disease [COVID-19]) vaccine, DNA, spike protein, adenovirus type 26 (Ad26) vector, preservative free, 5x1010 viral particles/0.5mL dosage, single dose*<br>● 0034A - Immunization administration by intramuscilar injection of severe acute respiratory syndrome coronavirys 2 (SARS-COV-2) (Coronavirus disease [COVID-19]) vaccine, DNA, spike protein, adenovirus type 26 (Ad26) vector, preservative free, SX1010 viral particles/0.5 mL dosage; booster dose<br>Any medical claim or pharmacy claim with one of the following NDC codes:<br>● 59676-580-05<br>● 59676-0580-05<br>● 59676-0580-15<br>● 59676-580-15<br>Any vaccination event with the following manufacturer description: JANSSEN, JANSSEN (DIVISION OF JOHNSON AND JOHNSON), JOHNSON AND JOHNSON, JSN, J&J, JNJ
Spikevax mRNA-1273 Vaccine (Moderna)	Any medical claim, pharmacy claim, inpatient hospital encounter, or outpatient hospital encounter with one of the following CPT codes:<br>● 91301 - Severe acute respiratory syndrome coronavirus 2 (SARS-CoV-2) (Coronavirus disease [COVID-19]) vaccine, mRNA-LNP, spike protein, preservative free, 100 mcg/0.5mL dosage, for intramuscular use<br>● 91306 - Severe acute respiratory syndrome coronavirus 2 (Sars-Cov-2) (Coronavirus disease [Covid-19]) vaccine, mRNA-LNP, spike protein, preservative free, 50 mcg/0.25 mL dosage, for intramuscular use<br>● 91309 - Severe acute respiratory syndrome coronavirus 2 (Sars-Cov-2) (Coronavirus disease [COVID-19]) vaccine, mRNA-LNP,, spike protein, preservative free, 50 mcg/0.5 mL dosage, for intramuscular use<br>● 91313 - Severe acute respiratory syndrome coronavirus 2 (Sars-Cov-2) (Coronavirus disease [COVID-19]) vaccine, mRNA-LNP,, spike protein, bivalent, preservative free, 50 mcg/0.5 mL dosage, for intramuscular use<br>● 91314 - Severe acute respiratory syndrome coronavirus 2 (Sars-Cov-2) (Coronavirus disease [COVID-19]) vaccine, mRNA-LNP,, spike protein, bivalent,

Variable name	Description
	preservative free, 25 mcg/0.25 mL dosage, for intramuscular use<br>● 0011A - Immunization administration by intramuscular injection of Severe acute respiratory syndrome coronavirus 2 (SARS-CoV-2) (Coronavirus disease [COVID-19]) vaccine, mRNA-LNP, spike protein, preservative free, 100 mcg/0.5mL dosage; first dose<br>● 0012A - Immunization administration by intramuscular injection of Severe acute respiratory syndrome coronavirus 2 (SARS-CoV-2) (Coronavirus disease [COVID-19]) vaccine, mRNA-LNP, spike protein, preservative free, 100 mcg/0.5mL dosage; second dose<br>● 0013A - Immunization administration by intramuscular injection of Severe acute respiratory syndrome coronavirus 2 (SARS-CoV-2) (Coronavirus disease [COVID-19]) vaccine, mRNA-LNP, spike protein, preservative free, 100 mcg/0.5 mL dosage; third dose<br>● 0064A - Immunization administration by intramuscular injection of Severe acute respiratory syndrome coronavirus 2 (SARS-CoV-2) (Coronavirus disease [COVID-19]) vaccine, mRNA-LNP, spike protein, preservative free, 50 mcg/0.25 mL dosage, booster dose<br>● 0094A - Immunization administration by intramuscular injection of Severe acute respiratory syndrome coronavirus 2 (SARS-CoV-2) (Coronavirus disease [COVID-19]) vaccine, mRNA-LNP, spike protein, preservative free, 50 mcg/0.5 mL dosage; booster dose, when administered to individuals 18 years and over<br>Any medical claim or pharmacy claim with one of the following NDC codes:<br>● 80777-0100-11<br>● 80777-0100-99<br>● 80777-0273-10<br>● 80777-0273-15<br>● 80777-0273-98<br>● 80777-0273-99<br>● 80777-0275-05<br>● 80777-0275-99<br>● 80777-0280-05<br>● 80777-0280-99<br>● 80777-0282-05<br>● 80777-273-10<br>● 80777-273-15<br>● 80777-273-98<br>● 80777-273-99<br>Any vaccination event with the following manufacturer description: MOD, MODERNA, MODERNA US, INC

Variable name	Description
Comirnaty BNT162b2 Vaccine (Pfizer)	Any medical claim, pharmacy claim, inpatient hospital encounter, or outpatient hospital encounter with one of the following CPT codes:<br>● 91300 - Severe acute respiratory syndrome coronavirus 2 (SARS-CoV-2) (Coronavirus disease [COVID-19]) vaccine, mRNA-LNP, spike protein, preservative free, 30 mcg/0.3mL dosage, diluent reconstituted, for intramuscular use<br>● 91305 - Severe acute respiratory syndrome coronavirus 2 (SARS-CoV-2) (Coronavirus disease [COVID-19]) vaccine, mRNA-LNP, spike protein, preservative free, 30 mcg/0.3 mL dosage, tris-sucrose formulation, for intramuscular use<br>● 91312 - Severe acute respiratory syndrome coronavirus 2 (SARS-CoV-2) (Coronavirus disease [COVID-19]) vaccine, mRNA-LNP, bivalent spike protein, preservative free, 30 mcg/0.3 ml dosage, tris-sucrose formulation, for intramuscular use<br>● 0001A - Immunization administration by intramuscular injection of severe acute respiratory syndrome coronavirus 2 (SARS-CoV-2) (Coronavirus disease [COVID-19]) vaccine, mRNA-LNP, spike protein, preservative free, 30 mcg/0.3mL dosage, diluent reconstituted; first dose<br>● 0002A - Immunization administration by intramuscular injection of severe acute respiratory syndrome coronavirus 2 (SARS-CoV-2) (Coronavirus disease [COVID-19]) vaccine, mRNA-LNP, spike protein, preservative free, 30 mcg/0.3mL dosage, diluent reconstituted; second dose<br>● 0003A - Immunization administration by intramuscular injection of severe acute respiratory syndrome coronavirus 2 (SARS-CoV-2) (Coronavirus disease [COVID-19]) vaccine, mRNA-LNP, spike protein, preservative free, 30 mcg/0.3 mL dosage, diluent reconstituted; third dose<br>● 0004A - Immunization administration by intramuscular injection of severe acute respiratory syndrome coronavirus 2 (SARS-CoV-2) (Coronavirus disease [COVID-19]) vaccine, mRNA-LNP, spike protein, preservative free, 30 mcg/0.3 mL dosage, diluent reconstituted; booster dose<br>● 0051A - Immunization administration by intramuscular injection of severe acute respiratory syndrome coronavirus 2 (SARS-CoV-2) (Coronavirus disease [COVID-19]) vaccine, mRNA-LNP, spike protein, preservative free, 30 mcg/0.3 mL dosage, tris-sucrose formulation; first dose<br>● 0052A - Immunization administration by intramuscular injection of severe acute respiratory syndrome coronavirus 2 (SARS-CoV-2) (Coronavirus disease [COVID-19]) vaccine, mRNA-LNP, spike protein, preservative free, 30 mcg/0.3 mL dosage, tris-sucrose formulation; second dose<br>● 0053A - Immunization administration by intramuscular injection of severe acute respiratory syndrome coronavirus 2 (SARS-CoV-2) (Coronavirus disease [COVID-19]) vaccine, mRNA-LNP, spike protein, preservative free, 30 mcg/0.3 mL dosage, tris-sucrose formulation; third dose<br>● 0054A - Immunization administration by intramuscular injection of severe acute

Variable name	Description
	respiratory syndrome coronavirus 2 (SARS-CoV-2) (Coronavirus disease [COVID-19]) vaccine, mRNA-LNP, spike protein, preservative free, 30 mcg/0.3 mL dosage, tris-sucrose formulation; booster dose<br>● 0124A - Immunization administration by intramuscular injection of severe acute respiratory syndrome coronavirus 2 (SARS-CoV-2) (Coronavirus disease [COVID-19]) vaccine, mRNA-LNP, bivalent spike protein, preservative free, 30 mcg/0.3 mL dosage, tris-sucrose formulation, booster dose<br>Any medical claim or pharmacy claim with one of the following NDC codes:<br>● 00069-2025-01<br>● 00069-2025-10<br>● 00069-2025-25<br>● 59267-0304-01<br>● 59267-0304-02<br>● 59267-1000-01<br>● 59267-1000-02<br>● 59267-1000-03<br>● 59267-100-01<br>● 59267-100-02<br>● 59267-1025-01<br>● 59267-1025-02<br>● 59267-1025-03<br>● 59267-1025-04<br>● 59267-102-51<br>● 59267-102-54<br>● 59267-105-54<br>● 59267-1404-01<br>● 59267-1404-02<br>● 05926-7100-03<br>Any vaccination event with the following manufacturer description: PFIZER, PFIZER, INC, PFR, PFI
Medically-attended COVID-19	Any diagnostic lab test with one of the following result values<br>● Positive<br><br>OR<br><br>Any medical claim, inpatient hospital encounter, or outpatient hospital encounter with one of the following ICD-10-CM codes: U07.1

Variable name	Description
Inpatient medical claim	Any medical claim with inpatient indicator or suspected inpatient indicator set to "Yes', or any inpatient hospital encounter
Occurrence of a COVID-19 related hospitalization	Any inpatient hospital medical claim or inpatient encounter for which any medically attended COVID-19 began in the 21 days prior to the start of the hospitalization through the last day of the hospitalization
COVID-19 related ICU admission	Any inpatient hospital medical claim or inpatient encounter for which any medically attended COVID-19 began in the 21 days prior to the start of the hospitalization through the last day of the hospitalization, and for which the hospitalization contains the following ICU-related revenue codes:<br>● 0201<br>● 0207<br>● 0209<br>● 0210<br>● 0211<br>● 0213<br>● 0214<br>● 0200<br>● 0202<br>● 0203<br>● 0204<br>● 0206<br>● 0208<br>● 0212<br>● 0219

Supplemental Table S2a: Baseline Characteristics, Pre PS Matching, for mRNA + Ad26.COV2.S vs Primary Series

Characteristic[a]	mRNA + Ad26.COV2.S vs mRNA + no boost			mRNA + Ad26.COV2.S vs Ad26.COV2.S + no boost		
N (%) or mean +/- SD unless otherwise noted	mRNA + Ad26.COV2.S	mRNA + no boost	ASD	mRNA + Ad26.COV2.S	Ad26.COV2.S + no boost	ASD
Number of Individuals	2,972	26,776		2,975	27,965	
Demographics						
Age, mean (SD)	46.90 (15.58)	46.54 (15.59)	0.023	46.89 (15.56)	46.85 (15.53)	0.003
Male sex; n (%)	1,584 (53.3%)	14,252 (53.2%)	0.001	1,589 (53.4%)	14,953 (53.5%)	0.001

U.S. Region[b]			0.022			0.013
...Northeast; n (%)	474 (15.9%)	4,428 (16.5%)		474 (15.9%)	4,579 (16.4%)	
...Midwest; n (%)	320 (10.8%)	2,896 (10.8%)		321 (10.8%)	2,999 (10.7%)	
...South; n (%)	650 (21.9%)	5,953 (22.2%)		651 (21.9%)	6,149 (22.0%)	
...West; n (%)	1,528 (51.4%)	13,499 (50.4%)		1,529 (51.4%)	14,238 (50.9%)	
State[c]			0.045			0.037
Index months			0.038			0.020
...October 2021; n (%)	127 (4.3%)	1,181 (4.4%)		127 (4.3%)	1,190 (4.3%)	
...November 2021; n (%)	877 (29.5%)	7,916 (29.6%)		876 (29.4%)	8,207 (29.3%)	
...December 2021; n (%)	1,169 (39.3%)	10,161 (37.9%)		1,171 (39.4%)	10,819 (38.7%)	
...January 2022; n (%)	403 (13.6%)	3,657 (13.7%)		406 (13.6%)	3,873 (13.8%)	
...February 2022 to September 2022; n (%)	396 (13.3%)	3861 (14.4%)		395 (13.3%)	3876 (13.9%)	
Commercial Enrollment on CED; n (%)	1,634 (55.0%)	13,463 (50.3%)	0.094	1,636 (55.0%)	15,036 (53.8%)	0.025
Medicare Advantage Enrollment on CED; n (%)	204 (6.9%)	2,013 (7.5%)	0.025	202 (6.8%)	2,025 (7.2%)	0.018
Medicaid Enrollment on CED; n (%)	1,248 (42.0%)	11,783 (44.0%)	0.041	1,249 (42.0%)	11,432 (40.9%)	0.022
COVID-19-related characteristics						
Receipt of any laboratory test for COVID-19; n (%)	833 (28.0%)	6,617 (24.7%)	0.075	835 (28.1%)	6,790 (24.3%)	0.086
History of COVID-19 infection; n (%)	269 (9.1%)	2,365 (8.8%)	0.008	270 (9.1%)	2,338 (8.4%)	0.025
Month of primary vaccination; n (%)[d]						
...January 2021; n (%)	91 (3.1%)	837 (3.1%)	-	91 (3.1%)	915 (3.3%)	-
... February 2021; n (%)	301 (10.1%)	2,515 (9.4%)	-	301 (10.1%)	2,611 (9.3%)	-
... March 2021; n (%)	392 (13.2%)	3,403 (12.7%)	-	392 (13.2%)	3,793 (13.6%)	-
... April 2021; n (%)	1,035 (34.8%)	8,908 (33.3%)	-	1,033 (34.7%)	9,537 (34.1%)	-
... May 2021; n (%)	753 (25.3%)	7,071 (26.4%)	-	756 (25.4%)	7,157 (25.6%)	-

... June 2021 to July 2022; n (%)	400 (13.5%)	4,042 (15.1%)	-	402 (13.5%)	3,952 (14.1%)	-
Days since primary series (SD)[a]	245.04 (52.95)	244.02 (54.65)	0.019	244.98 (52.81)	245.12 (54.12)	0.003
Comorbid conditions						
Cerebrovascular disease; n (%)	101 (3.4%)	930 (3.5%)	0.004	101 (3.4%)	882 (3.2%)	0.014
Chronic kidney disease (CKD); n (%)	122 (4.1%)	1,304 (4.9%)	0.037	122 (4.1%)	1,361 (4.9%)	0.037
Chronic obstructive pulmonary disease (COPD); n (%)	398 (13.4%)	2,920 (10.9%)	0.076	399 (13.4%)	3,128 (11.2%)	0.068
Cystic Fibrosis; n (%)	0 (0.0%)	1 (0.0%)	0.009	0 (0.0%)	4 (0.0%)	0.017
HIV; n (%)	33 (1.1%)	189 (0.7%)	0.043	33 (1.1%)	209 (0.7%)	0.038
Hypertension; n (%)	926 (31.2%)	7,972 (29.8%)	0.030	923 (31.0%)	8,554 (30.6%)	0.009
Immunocompromised state from organ transplant; n (%)	10 (0.3%)	132 (0.5%)	0.024	10 (0.3%)	192 (0.7%)	0.049
Immunocompromised state from blood transplant; n (%)	13 (0.4%)	131 (0.5%)	0.008	13 (0.4%)	156 (0.6%)	0.017
Liver disease; n (%)	205 (6.9%)	1,794 (6.7%)	0.008	207 (7.0%)	1,905 (6.8%)	0.006
Malignancies; n (%)	105 (3.5%)	963 (3.6%)	0.003	105 (3.5%)	1,064 (3.8%)	0.015
Moderate-to-severe asthma; n (%)	35 (1.2%)	294 (1.1%)	0.008	35 (1.2%)	343 (1.2%)	0.005
Neurologic Conditions; n (%)	953 (32.1%)	7,639 (28.5%)	0.077	955 (32.1%)	8,050 (28.8%)	0.072
Obesity; n (%)	732 (24.6%)	6,541 (24.4%)	0.005	730 (24.5%)	6,913 (24.7%)	0.004
Pulmonary fibrosis; n (%)	12 (0.4%)	170 (0.6%)	0.032	12 (0.4%)	152 (0.5%)	0.020
Serious heart conditions; n (%)	260 (8.7%)	2,211 (8.3%)	0.018	259 (8.7%)	2,365 (8.5%)	0.009
Sickle-cell disease; n (%)	0 (0.0%)	32 (0.1%)	0.049	0 (0.0%)	33 (0.1%)	0.049
Thalassemia; n (%)	2 (0.1%)	48 (0.2%)	0.032	2 (0.1%)	55 (0.2%)	0.036
Type 1 diabetes mellitus; n (%)	38 (1.3%)	304 (1.1%)	0.013	38 (1.3%)	330 (1.2%)	0.009

Type 2 diabetes mellitus; n (%)	446 (15.0%)	4,300 (16.1%)	0.029	444 (14.9%)	4,549 (16.3%)	0.037
Gagne combined comorbidity score, mean (SD)	0.80 (1.76)	0.78 (1.81)	0.011	0.80 (1.76)	0.77 (1.76)	0.019
Healthcare Resource Utilization						
Recent medical claim; n (%)[e]	2,020 (68.0%)	16,198 (60.5%)	0.156	2,019 (67.9%)	17,947 (64.2%)	0.078
Recent pharmacy claim; n (%)[e]	2,060 (69.3%)	16,897 (63.1%)	0.132	2,062 (69.3%)	19,158 (68.5%)	0.017

Abbreviations: ASD = absolute standardized difference; CKD = chronic kidney disease; COPD = chronic obstructive pulmonary disease; HIV = human immunodeficiency virus; ICU = intensive care unit; Ad26.COV2.S = Johnson and Johnson; mRNA = messenger ribonucleic acid; PS = propensity score; SD = standard deviation

[a] Characteristics reported for population matched with propensity scores. Unless otherwise noted, demographic variables are assessed at cohort entry (index) and comorbidities and clinical utilization variables are assessed during the 1 year before cohort entry.

[b] Variables excluded from final propensity score models, for overall and all stratified results.

[c] Individual state covariates are not shown for brevity.

[d] Month of primary vaccination refers to month of completion of either a single-dose Ad26.COV2.S primary vaccine series occurring any time between January 1st, 2021 and July 6th, 2022 (61 days prior to last possible index date) or a homologous two-dose mRNA primary vaccine series occurring between January 1st, 2021 and April 6th, 2022 (152 days prior to last possible index date). June 2021-July 2022 are presented as aggregated frequencies; ASDs are not reported.

[e] Recent medical and pharmacy claims were defined as claims beginning during the 60 days before cohort entry.

Supplemental Table S2b: Baseline Characteristics, Pre PS Matching, for Ad26.COV2.S + Ad26.COV2.S vs Primary Series

Characteristic[a]	Ad26.COV2.S + Ad26.COV2.S vs mRNA + no boost			Ad26.COV2.S + Ad26.COV2.S vs Ad26.COV2.S + no boost		
N (%) or mean +/- SD unless otherwise noted	Ad26.COV2.S + Ad26.COV2.S	mRNA + no boost	ASD	Ad26.COV2.S + Ad26.COV2.S	Ad26.COV2.S + no boost	ASD
Number of Individuals	74,628	668,503		43,078	389,305	
Demographics						
Age, mean (SD)	51.19 (14.93)	50.80 (15.04)	0.026	49.08 (15.46)	48.39 (15.14)	0.045
Male sex; n (%)	36,904 (49.5%)	329,989 (49.4%)	0.002	21,375 (49.6%)	193,635 (49.7%)	0.002
U.S. Region[b]			0.022			0.010
...Northeast; n (%)	14,866 (19.9%)	137,276 (20.5%)		8,838 (20.5%)	81,382 (20.9%)	

...Midwest; n (%)	12,125 (16.2%)	107,659 (16.1%)		7,593 (17.6%)	68,209 (17.5%)	
...South; n (%)	15,255 (20.4%)	139,648 (20.9%)		9,536 (22.1%)	85,957 (22.1%)	
...West; n (%)	32,382 (43.4%)	283,920 (42.5%)		17,111 (39.7%)	153,757 (39.5%)	
State[c]			0.029			0.048
Index months			0.038			0.014
...October 2021; n (%)	5,981 (8.0%)	54,027 (8.1%)		5,593 (13.0%)	49,742 (12.8%)	
...November 2021; n (%)	25,936 (34.8%)	227,050 (34.0%)		17,471 (40.6%)	157,111 (40.4%)	
...December 2021; n (%)	22,071 (29.6%)	192,351 (28.8%)		10,297 (23.9%)	92,609 (23.8%)	
...January 2022; n (%)	12,009 (16.1%)	110,863 (16.6%)		5,457 (12.7%)	50,055 (12.9%)	
... February 2022 to September 2022; n (%)	8631 (11.6%)	84212 (12.6%)		4260 (9.9%)	39788 (10.2%)	
Commercial Enrollment on CED; n (%)	38,657 (51.8%)	337,324 (50.5%)	0.027	22,412 (52.0%)	197,276 (50.7%)	0.027
Medicare Advantage Enrollment on CED; n (%)	6,748 (9.0%)	67,619 (10.1%)	0.036	3,594 (8.3%)	30,146 (7.7%)	0.022
Medicaid Enrollment on CED; n (%)	30,444 (40.8%)	276,269 (41.3%)	0.011	17,829 (41.4%)	168,602 (43.3%)	0.039
COVID-19-related characteristics						
Receipt of any laboratory test for COVID-19; n (%)	16,466 (22.1%)	156,333 (23.4%)	0.032	9,620 (22.3%)	86,254 (22.2%)	0.004
History of COVID-19 infection; n (%)	6,371 (8.5%)	59,763 (8.9%)	0.014	3,724 (8.6%)	38,971 (10.0%)	0.047
Month of primary vaccination; n (%)[d]						
...January 2021; n (%)	0 (0.0%)	0 (0.0%)	-	0 (0.0%)	0 (0.0%)	-
... February 2021; n (%)	1 (0.0%)	22,698 (3.4%)	-	0 (0.0%)	7 (0.0%)	-
... March 2021; n (%)	20,103 (26.9%)	159,825 (23.9%)	-	10,408 (24.2%)	87,868 (22.6%)	-
... April 2021; n (%)	29,642 (39.7%)	254,527 (38.1%)	-	15,884 (36.9%)	143,211 (36.8%)	-
... May 2021; n (%)	11,169 (15.0%)	96,643 (14.5%)	-	6,933 (16.1%)	66,126 (17.0%)	-
... June 2021 to July 2022; n (%)	13,713 (18.4%)	134,810 (20.2%)	-	9,853 (22.9%)	92,093 (23.7%)	-

Days since primary series (SD)[a]	230.89 (52.73)	228.63 (54.94)	0.042	215.98 (51.55)	215.02 (52.09)	0.019
Comorbid conditions						
Cerebrovascular disease; n (%)	2,599 (3.5%)	23,924 (3.6%)	0.005	1,438 (3.3%)	11,959 (3.1%)	0.015
Chronic kidney disease (CKD); n (%)	3,824 (5.1%)	33,060 (4.9%)	0.008	1,998 (4.6%)	14,707 (3.8%)	0.043
Chronic obstructive pulmonary disease (COPD); n (%)	9,521 (12.8%)	75,984 (11.4%)	0.043	5,461 (12.7%)	42,994 (11.0%)	0.051
Cystic Fibrosis; n (%)	2 (0.0%)	50 (0.0%)	0.007	1 (0.0%)	44 (0.0%)	0.011
HIV; n (%)	541 (0.7%)	3,917 (0.6%)	0.017	313 (0.7%)	2,454 (0.6%)	0.012
Hypertension; n (%)	27,665 (37.1%)	236,785 (35.4%)	0.034	14,906 (34.6%)	120,099 (30.8%)	0.080
Immunocompromised state from organ transplant; n (%)	387 (0.5%)	3,365 (0.5%)	0.002	226 (0.5%)	1,510 (0.4%)	0.020
Immunocompromised state from blood transplant; n (%)	398 (0.5%)	3,349 (0.5%)	0.005	211 (0.5%)	1,405 (0.4%)	0.020
Liver disease; n (%)	4,850 (6.5%)	43,696 (6.5%)	0.002	2,714 (6.3%)	22,921 (5.9%)	0.017
Malignancies; n (%)	3,016 (4.0%)	27,029 (4.0%)	0.000	1,641 (3.8%)	12,480 (3.2%)	0.033
Moderate-to-severe asthma; n (%)	1,013 (1.4%)	8,163 (1.2%)	0.012	606 (1.4%)	4,283 (1.1%)	0.028
Neurologic Conditions; n (%)	23,443 (31.4%)	200,065 (29.9%)	0.032	13,305 (30.9%)	110,918 (28.5%)	0.052
Obesity; n (%)	19,819 (26.6%)	172,593 (25.8%)	0.017	11,119 (25.8%)	90,068 (23.1%)	0.062
Pulmonary fibrosis; n (%)	442 (0.6%)	4,210 (0.6%)	0.005	247 (0.6%)	1,898 (0.5%)	0.012
Serious heart conditions; n (%)	6,927 (9.3%)	60,873 (9.1%)	0.006	3,699 (8.6%)	29,443 (7.6%)	0.038
Sickle-cell disease; n (%)	67 (0.1%)	647 (0.1%)	0.002	43 (0.1%)	331 (0.1%)	0.005
Thalassemia; n (%)	96 (0.1%)	1,093 (0.2%)	0.009	52 (0.1%)	477 (0.1%)	0.001
Type 1 diabetes mellitus; n (%)	831 (1.1%)	7,027 (1.1%)	0.006	457 (1.1%)	3,367 (0.9%)	0.020
Type 2 diabetes mellitus; n (%)	13,604 (18.2%)	119,430 (17.9%)	0.009	7,148 (16.6%)	55,703 (14.3%)	0.063

Gagne combined comorbidity score, mean (SD)	0.69 (1.66)	0.69 (1.67)	0.004	0.68 (1.64)	0.61 (1.54)	0.046
Healthcare Resource Utilization						
Recent medical claim; n (%)[e]	48,085 (64.4%)	422,389 (63.2%)	0.026	27,627 (64.1%)	230,779 (59.3%)	0.100
Recent pharmacy claim; n (%)[e]	53,039 (71.1%)	448,222 (67.0%)	0.087	30,199 (70.1%)	248,594 (63.9%)	0.133

Abbreviations: ASD = absolute standardized difference; CKD = chronic kidney disease; COPD = chronic obstructive pulmonary disease; HIV = human immunodeficiency virus; ICU = intensive care unit; Ad26.COV2.S = Johnson and Johnson; mRNA = messenger ribonucleic acid; PS = propensity score; SD = standard deviation

[a] Characteristics reported for population matched with propensity scores. Unless otherwise noted, demographic variables are assessed at cohort entry (index) and comorbidities and clinical utilization variables are assessed during the 1 year before cohort entry.

[b] Variables excluded from final propensity score models, for overall and all stratified results.

[c] Individual state covariates are not shown for brevity.

[d] Month of primary vaccination refers to month of completion of either a single-dose Ad26.COV2.S primary vaccine series occurring any time between January 1st, 2021 and July 6th, 2022 (61 days prior to last possible index date) or a homologous two-dose mRNA primary vaccine series occurring between January 1st, 2021 and April 6th, 2022 (152 days prior to last possible index date). June 2021-July 2022 are presented as aggregated frequencies; ASDs are not reported.

[e] Recent medical and pharmacy claims were defined as claims beginning during the 60 days before cohort entry.

Supplemental Table S3a. Baseline Characteristics, Pre PS Matching, for mRNA + Ad26.COV2.S vs Primary Series among Patients in the California State Registry

Characteristic[a]	mRNA + Ad26.COV2.S vs mRNA + no boost			mRNA + Ad26.COV2.S vs Ad26.COV2.S + no boost		
N (%) or mean +/- SD unless otherwise noted	mRNA + Ad26.COV2.S	mRNA + no boost	ASD	mRNA + Ad26.COV2.S	Ad26.COV2.S + no boost	ASD
Number of Individuals	1,193	11,107		1,195	11,831	
Demographics						
Age, mean (SD)	47.74 (15.60)	46.96 (15.94)	0.050	47.75 (15.58)	47.48 (15.93)	0.017
Male sex; n (%)	633 (53.1%)	5,837 (52.6%)	0.010	635 (53.1%)	6,295 (53.2%)	0.001
U.S. Region[b]			-			-
...Northeast; n (%)	0 (0.0%)	0 (0.0%)		0 (0.0%)	0 (0.0%)	
...Midwest; n (%)	0 (0.0%)	0 (0.0%)		0 (0.0%)	0 (0.0%)	
...South; n (%)	0 (0.0%)	0 (0.0%)		0 (0.0%)	0 (0.0%)	

...West; n (%)	1,193 (100.0%)	11,107 (100.0%)		1,195 (100.0%)	11,831 (100.0%)	
State[c]						
Index months			0.069			0.042
...October 2021; n (%)	52 (4.4%)	556 (5.0%)		52 (4.4%)	566 (4.8%)	
...November 2021; n (%)	359 (30.1%)	3,448 (31.0%)		358 (30.0%)	3,615 (30.6%)	
...December 2021; n (%)	439 (36.8%)	3,850 (34.7%)		441 (36.9%)	4,199 (35.5%)	
...January 2022; n (%)	183 (15.3%)	1,645 (14.8%)		184 (15.4%)	1,815 (15.3%)	
...February 2022 to September 2022; n (%)	160 (13.4%)	1608 (14.5%)		160 (13.4%)	1636 (13.8%)	
Commercial Enrollment on CED; n (%)	588 (49.3%)	4,752 (42.8%)	0.131	591 (49.5%)	5,750 (48.6%)	0.017
Medicare Advantage Enrollment on CED; n (%)	92 (7.7%)	873 (7.9%)	0.006	92 (7.7%)	955 (8.1%)	0.014
Medicaid Enrollment on CED; n (%)	541 (45.3%)	5,681 (51.1%)	0.116	540 (45.2%)	5,344 (45.2%)	0.000
COVID-19-related characteristics						
Receipt of any laboratory test for COVID-19; n (%)	323 (27.1%)	2,606 (23.5%)	0.083	325 (27.2%)	2,686 (22.7%)	0.104
History of COVID-19 infection; n (%)	93 (7.8%)	815 (7.3%)	0.017	94 (7.9%)	791 (6.7%)	0.045
Month of primary vaccination; n (%)[d]						
...January 2021; n (%)	44 (3.7%)	375 (3.4%)	-	44 (3.7%)	459 (3.9%)	-
... February 2021; n (%)	151 (12.7%)	1,282 (11.5%)	-	151 (12.6%)	1,382 (11.7%)	-
... March 2021; n (%)	196 (16.4%)	1,655 (14.9%)	-	196 (16.4%)	1,892 (16.0%)	-
... April 2021; n (%)	387 (32.4%)	3,643 (32.8%)	-	386 (32.3%)	3,927 (33.2%)	-
... May 2021; n (%)	275 (23.1%)	2,708 (24.4%)	-	276 (23.1%)	2,703 (22.8%)	-
... June 2021 to July 2022; n (%)	140 (11.7%)	1,444 (13%)	-	142 (11.9%)	1,468 (12.4%)	-
Days since primary series (SD)[a]	249.42 (51.13)	247.20 (55.00)	0.042	249.36 (50.97)	249.07 (54.55)	0.005
Comorbid conditions						

Cerebrovascular disease; n (%)	31 (2.6%)	337 (3.0%)	0.026	31 (2.6%)	285 (2.4%)	0.012
Chronic kidney disease (CKD); n (%)	56 (4.7%)	544 (4.9%)	0.010	56 (4.7%)	610 (5.2%)	0.022
Chronic obstructive pulmonary disease (COPD); n (%)	131 (11.0%)	1,003 (9.0%)	0.065	131 (11.0%)	1,052 (8.9%)	0.069
Cystic Fibrosis; n (%)	0 (0.0%)	0 (0.0%)	-	0 (0.0%)	2 (0.0%)	0.018
HIV; n (%)	7 (0.6%)	64 (0.6%)	0.001	7 (0.6%)	86 (0.7%)	0.017
Hypertension; n (%)	331 (27.7%)	2,957 (26.6%)	0.025	332 (27.8%)	3,328 (28.1%)	0.008
Immunocompromised state from organ transplant; n (%)	4 (0.3%)	60 (0.5%)	0.031	4 (0.3%)	95 (0.8%)	0.062
Immunocompromised state from blood transplant; n (%)	6 (0.5%)	80 (0.7%)	0.028	6 (0.5%)	98 (0.8%)	0.040
Liver disease; n (%)	73 (6.1%)	748 (6.7%)	0.025	74 (6.2%)	804 (6.8%)	0.024
Malignancies; n (%)	33 (2.8%)	363 (3.3%)	0.029	33 (2.8%)	451 (3.8%)	0.059
Moderate-to-severe asthma; n (%)	14 (1.2%)	98 (0.9%)	0.029	14 (1.2%)	108 (0.9%)	0.025
Neurologic Conditions; n (%)	359 (30.1%)	2,783 (25.1%)	0.113	359 (30.0%)	3,029 (25.6%)	0.099
Obesity; n (%)	265 (22.2%)	2,631 (23.7%)	0.035	264 (22.1%)	2,823 (23.9%)	0.042
Pulmonary fibrosis; n (%)	6 (0.5%)	59 (0.5%)	0.004	6 (0.5%)	54 (0.5%)	0.007
Serious heart conditions; n (%)	90 (7.5%)	813 (7.3%)	0.009	90 (7.5%)	875 (7.4%)	0.005
Sickle-cell disease; n (%)	0 (0.0%)	9 (0.1%)	0.040	0 (0.0%)	3 (0.0%)	0.023
Thalassemia; n (%)	0 (0.0%)	31 (0.3%)	0.075	0 (0.0%)	28 (0.2%)	0.069
Type 1 diabetes mellitus; n (%)	12 (1.0%)	112 (1.0%)	0.000	12 (1.0%)	119 (1.0%)	0.000
Type 2 diabetes mellitus; n (%)	164 (13.7%)	1,872 (16.9%)	0.086	164 (13.7%)	1,948 (16.5%)	0.077
Gagne combined comorbidity score, mean (SD)	0.74 (1.66)	0.71 (1.72)	0.019	0.74 (1.66)	0.69 (1.65)	0.030

Healthcare Resource Utilization						
Recent medical claim; n (%)[e]	777 (65.1%)	6,274 (56.5%)	0.178	778 (65.1%)	7,334 (62.0%)	0.065
Recent pharmacy claim; n (%)[e]	780 (65.4%)	6,647 (59.8%)	0.115	783 (65.5%)	7,728 (65.3%)	0.004

Abbreviations: ASD = absolute standardized difference; CKD = chronic kidney disease; COPD = chronic obstructive pulmonary disease; HIV = human immunodeficiency virus; ICU = intensive care unit; Ad26.COV2.S = Johnson and Johnson; mRNA = messenger ribonucleic acid; PS = propensity score; SD = standard deviation

[a] Characteristics reported for population matched with propensity scores. Unless otherwise noted, demographic variables are assessed at cohort entry (index) and comorbidities and clinical utilization variables are assessed during the 1 year before cohort entry.

[b] Variables excluded from final propensity score models, for overall and all stratified results.

[c] Individual state covariates are not shown for brevity.

[d] Month of primary vaccination refers to month of completion of either a single-dose Ad26.COV2.S primary vaccine series occurring any time between January 1st, 2021 and July 6th, 2022 (61 days prior to last possible index date) or a homologous two-dose mRNA primary vaccine series occurring between January 1st, 2021 and April 6th, 2022 (152 days prior to last possible index date). June 2021-July 2022 are presented as aggregated frequencies; ASDs are not reported.

[e] Recent medical and pharmacy claims were defined as claims beginning during the 60 days before cohort entry.

Supplemental Table S3b. Baseline Characteristics, Pre PS Matching, for Ad26.COV2.S + Ad26.COV2.S vs Primary Series among Patients in the California State Registry

Characteristic[a]	Ad26.COV2.S + Ad26.COV2.S vs mRNA + no boost			Ad26.COV2.S + Ad26.COV2.S vs Ad26.COV2.S + no boost		
N (%) or mean +/- SD unless otherwise noted	Ad26.COV2.S + Ad26.COV2.S	mRNA + no boost	ASD	Ad26.COV2.S + Ad26.COV2.S	Ad26.COV2.S + no boost	ASD
Number of Individuals	26,156	231,894		13,197	119,063	
Demographics						
Age, mean (SD)	51.41 (15.40)	50.74 (15.57)	0.043	48.84 (15.92)	48.19 (15.75)	0.041
Male sex; n (%)	12,934 (49.4%)	114,547 (49.4%)	0.001	6,569 (49.8%)	59,607 (50.1%)	0.006
U.S. Region[b]			-			-
...Northeast; n (%)	0 (0.0%)	0 (0.0%)		0 (0.0%)	0 (0.0%)	
...Midwest; n (%)	0 (0.0%)	0 (0.0%)		0 (0.0%)	0 (0.0%)	
...South; n (%)	0 (0.0%)	0 (0.0%)		0 (0.0%)	0 (0.0%)	
...West; n (%)	26,156 (100.0%)	231,894 (100.0%)		13,197 (100.0%)	119,063 (100.0%)	

Characteristic[a]	Ad26.COV2.S + Ad26.COV2.S vs mRNA + no boost			Ad26.COV2.S + Ad26.COV2.S vs Ad26.COV2.S + no boost		
N (%) or mean +/- SD unless otherwise noted	Ad26.COV2.S + Ad26.COV2.S	mRNA + no boost	ASD	Ad26.COV2.S + Ad26.COV2.S	Ad26.COV2.S + no boost	ASD
State[c]						
Index months			0.053			0.023
...October 2021; n (%)	2,076 (7.9%)	19,059 (8.2%)		1,938 (14.7%)	17,243 (14.5%)	
...November 2021; n (%)	9,440 (36.1%)	81,903 (35.3%)		5,828 (44.2%)	51,793 (43.5%)	
...December 2021; n (%)	7,060 (27.0%)	59,345 (25.6%)		2,570 (19.5%)	23,246 (19.5%)	
...January 2022; n (%)	4,228 (16.2%)	38,491 (16.6%)		1,554 (11.8%)	14,354 (12.1%)	
...February 2022 to September 2022; n (%)	3352 (12.8%)	33096 (14.3%)		1307 (9.9%)	12427 (10.4%)	
Commercial Enrollment on CED; n (%)	11,458 (43.8%)	98,890 (42.6%)	0.023	5,856 (44.4%)	49,397 (41.5%)	0.058
Medicare Advantage Enrollment on CED; n (%)	2,605 (10.0%)	22,450 (9.7%)	0.009	1,131 (8.6%)	8,811 (7.4%)	0.043
Medicaid Enrollment on CED; n (%)	12,514 (47.8%)	114,719 (49.5%)	0.033	6,436 (48.8%)	62,885 (52.8%)	0.081
COVID-19-related characteristics						
Receipt of any laboratory test for COVID-19; n (%)	5,949 (22.7%)	51,439 (22.2%)	0.013	3,035 (23.0%)	26,043 (21.9%)	0.027
History of COVID-19 infection; n (%)	2,033 (7.8%)	17,968 (7.7%)	0.001	1,025 (7.8%)	10,402 (8.7%)	0.035
Month of primary vaccination; n (%)[d]						
...January 2021; n (%)	0 (0.0%)	0 (0.0%)	-	0 (0.0%)	0 (0.0%)	-
... February 2021; n (%)	1 (0.0%)	6,699 (2.9%)	0.243	0 (0.0%)	0 (0.0%)	-
... March 2021; n (%)	7,685 (29.4%)	64,741 (27.9%)	0.032	3,554 (26.9%)	29,919 (25.1%)	0.041
... April 2021; n (%)	11,332 (43.3%)	91,385 (39.4%)	0.080	5,265 (39.9%)	46,823 (39.3%)	0.012
... May 2021; n (%)	2,598 (9.9%)	24,323 (10.5%)	0.018	1,436 (10.9%)	13,647 (11.5%)	0.018
... June 2021 to July 2022; n (%)	4,540 (17.4%)	44,746 (19.3%)	-	2,942 (22.3%)	28,674 (24.1%)	-

Characteristic[a]	Ad26.COV2.S + Ad26.COV2.S vs mRNA + no boost			Ad26.COV2.S + Ad26.COV2.S vs Ad26.COV2.S + no boost		
N (%) or mean +/- SD unless otherwise noted	Ad26.COV2.S + Ad26.COV2.S	mRNA + no boost	ASD	Ad26.COV2.S + Ad26.COV2.S	Ad26.COV2.S + no boost	ASD
Days since primary series (SD)[a]	234.08 (54.09)	231.11 (56.99)	0.053	215.24 (52.28)	213.28 (53.31)	0.037
Comorbid conditions						
Cerebrovascular disease; n (%)	785 (3.0%)	7,118 (3.1%)	0.004	363 (2.8%)	3,254 (2.7%)	0.001
Chronic kidney disease (CKD); n (%)	1,535 (5.9%)	13,288 (5.7%)	0.006	652 (4.9%)	5,151 (4.3%)	0.029
Chronic obstructive pulmonary disease (COPD); n (%)	2,758 (10.5%)	21,633 (9.3%)	0.041	1,339 (10.1%)	11,168 (9.4%)	0.026
Cystic Fibrosis; n (%)	0 (0.0%)	12 (0.0%)	0.010	0 (0.0%)	12 (0.0%)	0.014
HIV; n (%)	237 (0.9%)	1,316 (0.6%)	0.040	119 (0.9%)	833 (0.7%)	0.023
Hypertension; n (%)	8,795 (33.6%)	74,840 (32.3%)	0.029	3,914 (29.7%)	32,712 (27.5%)	0.048
Immunocompromised state from organ transplant; n (%)	149 (0.6%)	1,303 (0.6%)	0.001	74 (0.6%)	503 (0.4%)	0.020
Immunocompromised state from blood transplant; n (%)	205 (0.8%)	1,798 (0.8%)	0.001	96 (0.7%)	666 (0.6%)	0.021
Liver disease; n (%)	1,796 (6.9%)	15,980 (6.9%)	0.001	853 (6.5%)	7,431 (6.2%)	0.009
Malignancies; n (%)	967 (3.7%)	8,591 (3.7%)	0.000	462 (3.5%)	3,593 (3.0%)	0.027
Moderate-to-severe asthma; n (%)	286 (1.1%)	2,277 (1.0%)	0.011	138 (1.0%)	1,040 (0.9%)	0.018
Neurologic Conditions; n (%)	7,230 (27.6%)	61,028 (26.3%)	0.030	3,473 (26.3%)	30,094 (25.3%)	0.024
Obesity; n (%)	6,699 (25.6%)	58,773 (25.3%)	0.006	3,188 (24.2%)	26,824 (22.5%)	0.038
Pulmonary fibrosis; n (%)	169 (0.6%)	1,359 (0.6%)	0.008	86 (0.7%)	544 (0.5%)	0.026
Serious heart conditions; n (%)	2,144 (8.2%)	18,665 (8.0%)	0.005	909 (6.9%)	8,008 (6.7%)	0.006
Sickle-cell disease; n (%)	17 (0.1%)	110 (0.0%)	0.007	13 (0.1%)	74 (0.1%)	0.013

Characteristic[a]	Ad26.COV2.S + Ad26.COV2.S vs mRNA + no boost			Ad26.COV2.S + Ad26.COV2.S vs Ad26.COV2.S + no boost		
N (%) or mean +/- SD unless otherwise noted	Ad26.COV2.S + Ad26.COV2.S	mRNA + no boost	ASD	Ad26.COV2.S + Ad26.COV2.S	Ad26.COV2.S + no boost	ASD
Thalassemia; n (%)	44 (0.2%)	428 (0.2%)	0.004	22 (0.2%)	183 (0.2%)	0.003
Type 1 diabetes mellitus; n (%)	262 (1.0%)	2,144 (0.9%)	0.008	119 (0.9%)	919 (0.8%)	0.014
Type 2 diabetes mellitus; n (%)	4,947 (18.9%)	43,707 (18.8%)	0.002	2,172 (16.5%)	17,564 (14.8%)	0.047
Gagne combined comorbidity score, mean (SD)	0.70 (1.67)	0.69 (1.69)	0.009	0.65 (1.59)	0.64 (1.59)	0.010
Healthcare Resource Utilization						
Recent medical claim; n (%)[e]	16,294 (62.3%)	137,934 (59.5%)	0.058	8,158 (61.8%)	66,993 (56.3%)	0.113
Recent pharmacy claim; n (%)[e]	17,281 (66.1%)	147,992 (63.8%)	0.047	8,441 (64.0%)	70,325 (59.1%)	0.101

Abbreviations: ASD = absolute standardized difference; CKD = chronic kidney disease; COPD = chronic obstructive pulmonary disease; HIV = human immunodeficiency virus; ICU = intensive care unit; Ad26.COV2.S = Johnson and Johnson; mRNA = messenger ribonucleic acid; PS = propensity score; SD = standard deviation

[a] Characteristics reported for population matched with propensity scores. Unless otherwise noted, demographic variables are assessed at cohort entry (index) and comorbidities and clinical utilization variables are assessed during the 1 year before cohort entry.

[b] Variables excluded from final propensity score models, for overall and all stratified results.

[c] Individual state covariates are not shown for brevity.

[d] Month of primary vaccination refers to month of completion of either a single-dose Ad26.COV2.S primary vaccine series occurring any time between January 1st, 2021 and July 6th, 2022 (61 days prior to last possible index date) or a homologous two-dose mRNA primary vaccine series occurring between January 1st, 2021 and April 6th, 2022 (152 days prior to last possible index date). June 2021-July 2022 are presented as aggregated frequencies; ASDs are not reported.

[e] Recent medical and pharmacy claims were defined as claims beginning during the 60 days before cohort entry.

Supplemental Table S4a. Baseline Characteristics, Post PS Matching, for mRNA + Ad26.COV2.S vs Primary Series among Patients in the California State Registry

Characteristic[a]	mRNA + Ad26.COV2.S vs mRNA + no boost			mRNA + Ad26.COV2.S vs Ad26.COV2.S + no boost		
N (%) or mean +/- SD unless otherwise noted	mRNA + Ad26.COV2.S	mRNA + no boost	ASD	mRNA + Ad26.COV2.S	Ad26.COV2.S + no boost	ASD
Number of Individuals	1,192	4,631		1,193	4,622	
Demographics						
Age, mean (SD)	47.73 (15.61)	47.93 (15.66)	0.013	47.75 (15.59)	47.68 (15.72)	0.005
Male sex; n (%)	633 (53.1%)	2,408 (52.0%)	0.022	635 (53.2%)	2,493 (53.9%)	0.014
U.S. Region[b]			-			-
...Northeast; n (%)	0 (0.0%)	0 (0.0%)		0 (0.0%)	0 (0.0%)	
...Midwest; n (%)	0 (0.0%)	0 (0.0%)		0 (0.0%)	0 (0.0%)	
...South; n (%)	0 (0.0%)	0 (0.0%)		0 (0.0%)	0 (0.0%)	
...West; n (%)	1,192 (100.0%)	4,631 (100.0%)		1,193 (100.0%)	4,622 (100.0%)	
State[c]						
Index months			0.030			0.037
...October 2021; n (%)	52 (4.4%)	210 (4.5%)		52 (4.4%)	206 (4.5%)	
...November 2021; n (%)	359 (30.1%)	1,413 (30.5%)		358 (30.0%)	1,379 (29.8%)	
...December 2021; n (%)	439 (36.8%)	1,692 (36.5%)		441 (37.0%)	1,702 (36.8%)	
...January 2022; n (%)	183 (15.4%)	694 (15.0%)		184 (15.4%)	736 (15.9%)	
...February 2022 to September 2022; n (%)	159 (13.3%)	622 (13.4%)		158 (13.2%)	599 (13%)	
Commercial Enrollment on CED; n (%)	588 (49.3%)	2,250 (48.6%)	0.015	590 (49.5%)	2,317 (50.1%)	0.013
Medicare Advantage Enrollment on CED; n (%)	92 (7.7%)	370 (8.0%)	0.010	92 (7.7%)	306 (6.6%)	0.042
Medicaid Enrollment on CED; n (%)	540 (45.3%)	2,110 (45.6%)	0.005	539 (45.2%)	2,092 (45.3%)	0.002
COVID-19-related characteristics						
Receipt of any laboratory test for	323 (27.1%)	1,267 (27.4%)	0.006	324 (27.2%)	1,212 (26.2%)	0.021

COVID-19; n (%)						
History of COVID-19 infection; n (%)	93 (7.8%)	379 (8.2%)	0.014	94 (7.9%)	312 (6.8%)	0.043
Month of primary vaccination; n (%)[d]						
...January 2021; n (%)	44 (3.7%)	171 (3.7%)	-	44 (3.7%)	171 (3.7%)	-
... February 2021; n (%)	151 (12.7%)	576 (12.4%)	-	151 (12.7%)	539 (11.7%)	-
... March 2021; n (%)	196 (16.4%)	760 (16.4%)	-	196 (16.4%)	739 (16.0%)	-
... April 2021; n (%)	387 (32.5%)	1,528 (33.0%)	-	386 (32.4%)	1,492 (32.3%)	-
... May 2021; n (%)	275 (23.1%)	1,050 (22.7%)	-	276 (23.1%)	1,140 (24.7%)	-
... June 2021 to July 2022; n (%)	139 (11.7%)	546 (11.8%)	-	140 (11.7%)	541 (11.7%)	-
Days since primary series (SD)[a]	249.45 (51.14)	248.82 (53.98)	0.012	249.37 (50.99)	248.67 (54.45)	0.013
Comorbid conditions						
Cerebrovascular disease; n (%)	31 (2.6%)	130 (2.8%)	0.013	31 (2.6%)	116 (2.5%)	0.006
Chronic kidney disease (CKD); n (%)	56 (4.7%)	228 (4.9%)	0.011	56 (4.7%)	210 (4.5%)	0.007
Chronic obstructive pulmonary disease (COPD); n (%)	130 (10.9%)	507 (10.9%)	0.001	130 (10.9%)	481 (10.4%)	0.016
Cystic Fibrosis; n (%)	0 (0.0%)	0 (0.0%)	-	0 (0.0%)	0 (0.0%)	-
HIV; n (%)	7 (0.6%)	28 (0.6%)	0.002	7 (0.6%)	21 (0.5%)	0.018
Hypertension; n (%)	331 (27.8%)	1,281 (27.7%)	0.002	332 (27.8%)	1,259 (27.2%)	0.013
Immunocompromised state from organ transplant; n (%)	4 (0.3%)	22 (0.5%)	0.022	4 (0.3%)	21 (0.5%)	0.019
Immunocompromised state from blood transplant; n (%)	6 (0.5%)	29 (0.6%)	0.016	6 (0.5%)	25 (0.5%)	0.005
Liver disease; n (%)	73 (6.1%)	267 (5.8%)	0.015	74 (6.2%)	276 (6.0%)	0.010
Malignancies; n (%)	33 (2.8%)	140 (3.0%)	0.015	33 (2.8%)	128 (2.8%)	0.000
Moderate-to-severe asthma; n (%)	14 (1.2%)	46 (1.0%)	0.017	14 (1.2%)	58 (1.3%)	0.007

Neurologic Conditions; n (%)	358 (30.0%)	1,380 (29.8%)	0.005	358 (30.0%)	1,349 (29.2%)	0.018
Obesity; n (%)	265 (22.2%)	1,028 (22.2%)	0.001	264 (22.1%)	974 (21.1%)	0.026
Pulmonary fibrosis; n (%)	6 (0.5%)	27 (0.6%)	0.011	6 (0.5%)	23 (0.5%)	0.001
Serious heart conditions; n (%)	90 (7.6%)	341 (7.4%)	0.007	90 (7.5%)	331 (7.2%)	0.015
Sickle-cell disease; n (%)	0 (0.0%)	0 (0.0%)	-	0 (0.0%)	0 (0.0%)	-
Thalassemia; n (%)	0 (0.0%)	0 (0.0%)	-	0 (0.0%)	0 (0.0%)	-
Type 1 diabetes mellitus; n (%)	12 (1.0%)	53 (1.1%)	0.013	12 (1.0%)	44 (1.0%)	0.005
Type 2 diabetes mellitus; n (%)	164 (13.8%)	658 (14.2%)	0.013	164 (13.7%)	590 (12.8%)	0.029
Gagne combined comorbidity score, mean (SD)	0.74 (1.66)	0.74 (1.75)	0.001	0.74 (1.66)	0.69 (1.63)	0.028
Healthcare Resource Utilization						
Recent medical claim; n (%)[e]	776 (65.1%)	3,063 (66.1%)	0.022	776 (65.0%)	2,994 (64.8%)	0.006
Recent pharmacy claim; n (%)[e]	779 (65.4%)	3,003 (64.8%)	0.011	781 (65.5%)	3,014 (65.2%)	0.005

Supplemental Table S4b. Baseline Characteristics, Post PS Matching, for Ad26.COV2.S + Ad26.COV2.S vs Primary Series among Patients in the California State Registry

Characteristic[a]	Ad26.COV2.S + Ad26.COV2.S vs mRNA + no boost			Ad26.COV2.S + Ad26.COV2.S vs Ad26.COV2.S + no boost		
N (%) or mean +/- SD unless otherwise noted	Ad26.COV2.S + Ad26.COV2.S	mRNA + no boost	ASD	Ad26.COV2.S + Ad26.COV2.S	Ad26.COV2.S + no boost	ASD
Number of Individuals	26,156	101,115		13,197	51,202	
Demographics						
Age, mean (SD)	51.41 (15.40)	51.37 (15.28)	0.003	48.84 (15.92)	48.86 (15.67)	0.001
Male sex; n (%)	12,934 (49.4%)	50,159 (49.6%)	0.003	6,569 (49.8%)	25,427 (49.7%)	0.002
U.S. Region[b]			-			-
...Northeast; n (%)	0 (0.0%)	0 (0.0%)		0 (0.0%)	0 (0.0%)	

...Midwest; n (%)	0 (0.0%)	0 (0.0%)		0 (0.0%)	0 (0.0%)	
...South; n (%)	0 (0.0%)	0 (0.0%)		0 (0.0%)	0 (0.0%)	
...West; n (%)	26,156 (100.0%)	101,115 (100.0%)		13,197 (100.0%)	51,202 (100.0%)	
State[c]						
Index months			0.016			0.021
...October 2021; n (%)	2,076 (7.9%)	8,143 (8.1%)		1,938 (14.7%)	7,416 (14.5%)	
...November 2021; n (%)	9,440 (36.1%)	36,633 (36.2%)		5,828 (44.2%)	23,049 (45.0%)	
...December 2021; n (%)	7,060 (27.0%)	27,070 (26.8%)		2,570 (19.5%)	9,931 (19.4%)	
...January 2022; n (%)	4,228 (16.2%)	16,639 (16.5%)		1,554 (11.8%)	5,909 (11.5%)	
... February 2022 to September 2022; n (%)	3352 (12.8%)	12630 (12.5%)		1307 (9.9%)	4897 (9.6%)	
Commercial Enrollment on CED; n (%)	11,458 (43.8%)	44,704 (44.2%)	0.008	5,856 (44.4%)	22,791 (44.5%)	0.003
Medicare Advantage Enrollment on CED; n (%)	2,605 (10.0%)	9,539 (9.4%)	0.018	1,131 (8.6%)	4,294 (8.4%)	0.007
Medicaid Enrollment on CED; n (%)	12,514 (47.8%)	48,424 (47.9%)	0.001	6,436 (48.8%)	24,913 (48.7%)	0.002
COVID-19-related characteristics						
Receipt of any laboratory test for COVID-19; n (%)	5,949 (22.7%)	22,260 (22.0%)	0.018	3,035 (23.0%)	11,652 (22.8%)	0.006
History of COVID-19 infection; n (%)	2,033 (7.8%)	7,494 (7.4%)	0.014	1,025 (7.8%)	3,882 (7.6%)	0.007
Month of primary vaccination; n (%)[d]						
...January 2021; n (%)	0 (0.0%)	0 (0.0%)	-	0 (0.0%)	0 (0.0%)	-
... February 2021; n (%)	1 (0.0%)	4 (0.0%)	-	0 (0.0%)	0 (0.0%)	-
... March 2021; n (%)	7,685 (29.4%)	29,353 (29.0%)	-	3,554 (26.9%)	13,948 (27.2%)	-
... April 2021; n (%)	11,332 (43.3%)	44,382 (43.9%)	-	5,265 (39.9%)	20,615 (40.3%)	-
... May 2021; n (%)	2,598 (9.9%)	10,246 (10.1%)	-	1,436 (10.9%)	5,491 (10.7%)	-
... June 2021 to July 2022; n (%)	4,540 (17.4%)	17,130 (16.9%)	-	2,942 (22.3%)	11,148 (21.8%)	-

Days since primary series (SD)[a]	234.08 (54.09)	231.46 (53.94)	0.048	215.24 (52.28)	215.44 (51.93)	0.004
Comorbid conditions						
Cerebrovascular disease; n (%)	785 (3.0%)	2,973 (2.9%)	0.004	363 (2.8%)	1,374 (2.7%)	0.004
Chronic kidney disease (CKD); n (%)	1,535 (5.9%)	5,662 (5.6%)	0.012	652 (4.9%)	2,461 (4.8%)	0.006
Chronic obstructive pulmonary disease (COPD); n (%)	2,758 (10.5%)	10,150 (10.0%)	0.017	1,339 (10.1%)	5,022 (9.8%)	0.011
Cystic Fibrosis; n (%)	0 (0.0%)	0 (0.0%)	-	0 (0.0%)	0 (0.0%)	-
HIV; n (%)	237 (0.9%)	853 (0.8%)	0.007	119 (0.9%)	472 (0.9%)	0.002
Hypertension; n (%)	8,795 (33.6%)	32,973 (32.6%)	0.022	3,914 (29.7%)	14,934 (29.2%)	0.011
Immunocompromised state from organ transplant; n (%)	149 (0.6%)	594 (0.6%)	0.002	74 (0.6%)	291 (0.6%)	0.001
Immunocompromised state from blood transplant; n (%)	205 (0.8%)	799 (0.8%)	0.001	96 (0.7%)	359 (0.7%)	0.003
Liver disease; n (%)	1,796 (6.9%)	6,673 (6.6%)	0.011	853 (6.5%)	3,180 (6.2%)	0.010
Malignancies; n (%)	967 (3.7%)	3,579 (3.5%)	0.008	462 (3.5%)	1,720 (3.4%)	0.008
Moderate-to-severe asthma; n (%)	286 (1.1%)	1,062 (1.1%)	0.004	138 (1.0%)	527 (1.0%)	0.002
Neurologic Conditions; n (%)	7,230 (27.6%)	26,988 (26.7%)	0.021	3,473 (26.3%)	13,029 (25.4%)	0.020
Obesity; n (%)	6,699 (25.6%)	25,090 (24.8%)	0.018	3,188 (24.2%)	12,020 (23.5%)	0.016
Pulmonary fibrosis; n (%)	169 (0.6%)	606 (0.6%)	0.006	86 (0.7%)	340 (0.7%)	0.002
Serious heart conditions; n (%)	2,144 (8.2%)	7,904 (7.8%)	0.014	909 (6.9%)	3,425 (6.7%)	0.008
Sickle-cell disease; n (%)	17 (0.1%)	64 (0.1%)	0.001	13 (0.1%)	50 (0.1%)	0.000
Thalassemia; n (%)	44 (0.2%)	175 (0.2%)	0.001	22 (0.2%)	82 (0.2%)	0.002
Type 1 diabetes mellitus; n (%)	262 (1.0%)	949 (0.9%)	0.006	119 (0.9%)	439 (0.9%)	0.005
Type 2 diabetes mellitus; n (%)	4,947 (18.9%)	18,624 (18.4%)	0.013	2,172 (16.5%)	8,274 (16.2%)	0.008

Gagne combined comorbidity score, mean (SD)	0.70 (1.67)	0.67 (1.67)	0.020	0.65 (1.59)	0.63 (1.57)	0.017
Healthcare Resource Utilization						
Recent medical claim; n (%)[e]	16,294 (62.3%)	62,161 (61.5%)	0.017	8,158 (61.8%)	31,146 (60.8%)	0.020
Recent pharmacy claim; n (%)[e]	17,281 (66.1%)	66,229 (65.5%)	0.012	8,441 (64.0%)	32,463 (63.4%)	0.012

Abbreviations: ASD = absolute standardized difference; CKD = chronic kidney disease; COPD = chronic obstructive pulmonary disease; HIV = human immunodeficiency virus; ICU = intensive care unit; Ad26.COV2.S = Johnson and Johnson; mRNA = messenger ribonucleic acid; PS = propensity score; SD = standard deviation

[a] Characteristics reported for population matched with propensity scores. Unless otherwise noted, demographic variables are assessed at cohort entry (index) and comorbidities and clinical utilization variables are assessed during the 1 year before cohort entry.

[b] Variables excluded from final propensity score models, for overall and all stratified results.

[c] Individual state covariates are not shown for brevity.

[d] Month of primary vaccination refers to month of completion of either a single-dose Ad26.COV2.S primary vaccine series occurring any time between January 1st, 2021 and July 6th, 2022 (61 days prior to last possible index date) or a homologous two-dose mRNA primary vaccine series occurring between January 1st, 2021 and April 6th, 2022 (152 days prior to last possible index date). June 2021-July 2022 are presented as aggregated frequencies; ASDs are not reported.

[e] Recent medical and pharmacy claims were defined as claims beginning during the 60 days before cohort entry.

Supplemental Table S5. Adjusted Hazard Ratios for COVID-19-related Hospitalization and Medically attended COVID-19 among Patients in the California State Registry

	N events	Person- years	Incidence rate (per 1,000 person-years)	Median days follow-up [IQR]	Fully Adjusted HR (95% CI)*
mRNA + Ad26.COV2.S vs mRNA + no boost					
COVID-19-related hospitalization					
mRNA + Ad26.COV2.S	9	748	12.03	267 [170, 302]	0.83 (0.39, 1.74)
mRNA + no boost (ref)	30	1,861	16.12	117 [25, 274]	
Medically attended COVID-19					
mRNA + Ad26.COV2.S	89	720	123.57	258 [154, 300]	0.89 (0.70, 1.14)
mRNA + no boost (ref)	256	1,767	144.88	91 [23, 269]	
mRNA + Ad26.COV2.S vs Ad26.COV2.S + no boost					
COVID-19-related hospitalization					

mRNA + Ad26.COV2.S	9	749	12.01	267 [171, 302]	0.82 (0.39, 1.72)
Ad26.COV2.S + no boost (ref)	32	2,191	14.61	188 [47, 291]	
Medically attended COVID-19					
mRNA + Ad26.COV2.S	90	721	124.82	258 [154, 300]	0.95 (0.75, 1.21)
Ad26.COV2.S + no boost (ref)	282	2,090	134.90	170 [41, 281]	
Ad26.COV2.S + Ad26.COV2.S vs mRNA + no boost					
COVID-19-related hospitalization					
Ad26.COV2.S + Ad26.COV2.S	183	17,658	10.36	273 [202, 310]	0.77 (0.65, 0.91)
mRNA + no boost (ref)	592	39,869	14.85	99 [24, 270]	
Medically attended COVID-19					
Ad26.COV2.S + Ad26.COV2.S	1,998	16,854	118.55	266 [180, 308]	0.96 (0.91, 1.01)
mRNA + no boost (ref)	5,078	37,981	133.70	82 [23, 264]	
Ad26.COV2.S + Ad26.COV2.S vs Ad26.COV2.S + no boost					
COVID-19-related hospitalization					
Ad26.COV2.S + Ad26.COV2.S	86	9,014	9.54	282 [195, 322]	0.54 (0.43, 0.68)
Ad26.COV2.S + no boost (ref)	427	23,100	18.48	159 [33, 300]	
Medically attended COVID-19					
Ad26.COV2.S + Ad26.COV2.S	1,008	8,599	117.23	271 [175, 320]	0.88 (0.82, 0.95)
Ad26.COV2.S + no boost (ref)	3,066	21,944	139.72	125 [31, 292]	

Abbreviations: CI = confidence interval; HR = hazard ratio; IQR = interquartile range; Ad26.COV2.S = Johnson and Johnson; mRNA = messenger ribonucleic acid

* Fully adjusted hazard ratio refers to the hazard ratio obtained from a Cox Proportional Hazards Model comparing study groups that were balanced using PS matching. For study groups that remained imbalanced after PS matching (absolute standardized mean difference ≥0.1 for a covariate), a doubly-robust adjustment was applied by adding those imbalanced covariates to the outcome model.

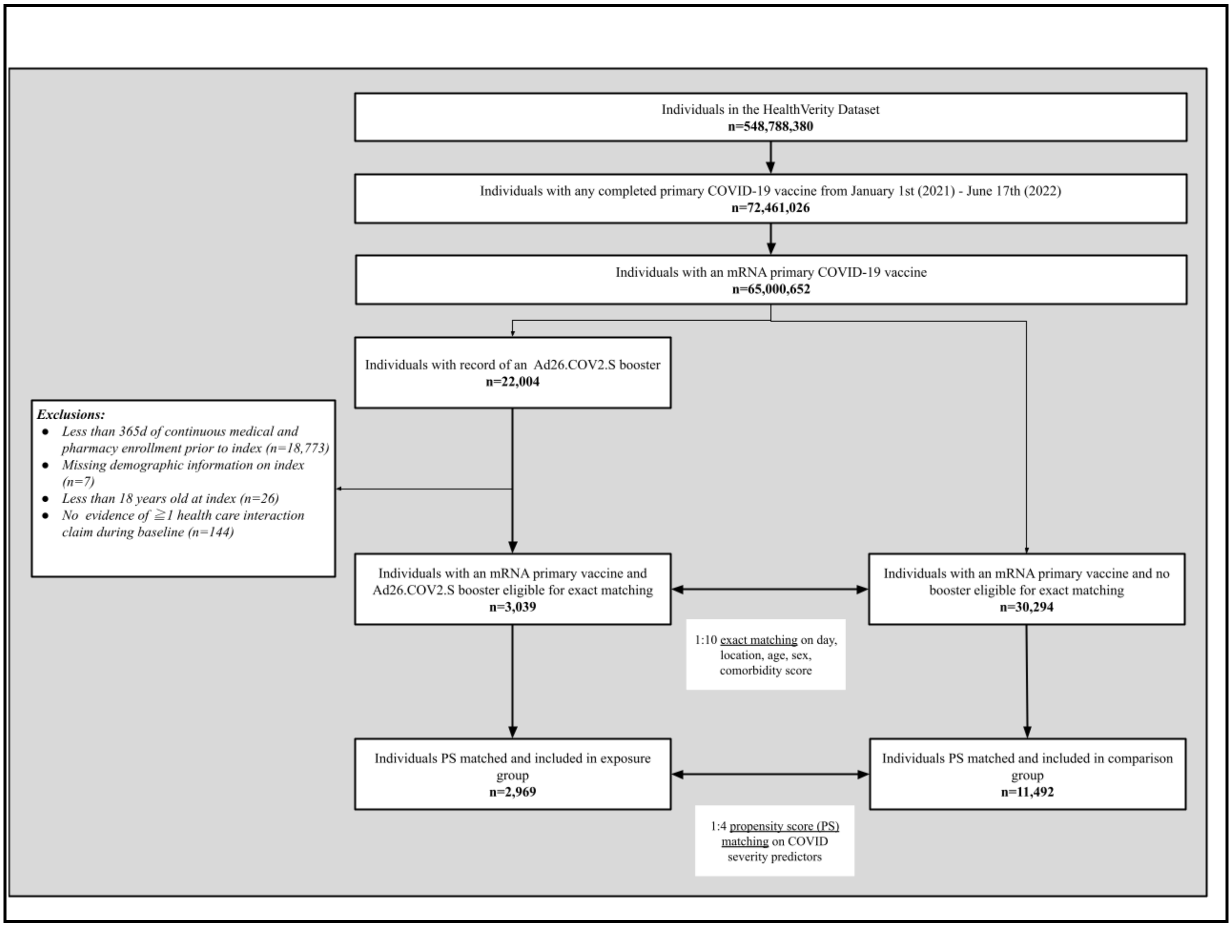

Figure S1. Study population flow diagram, mRNA + Ad26.COV2.S vs mRNA + no boost

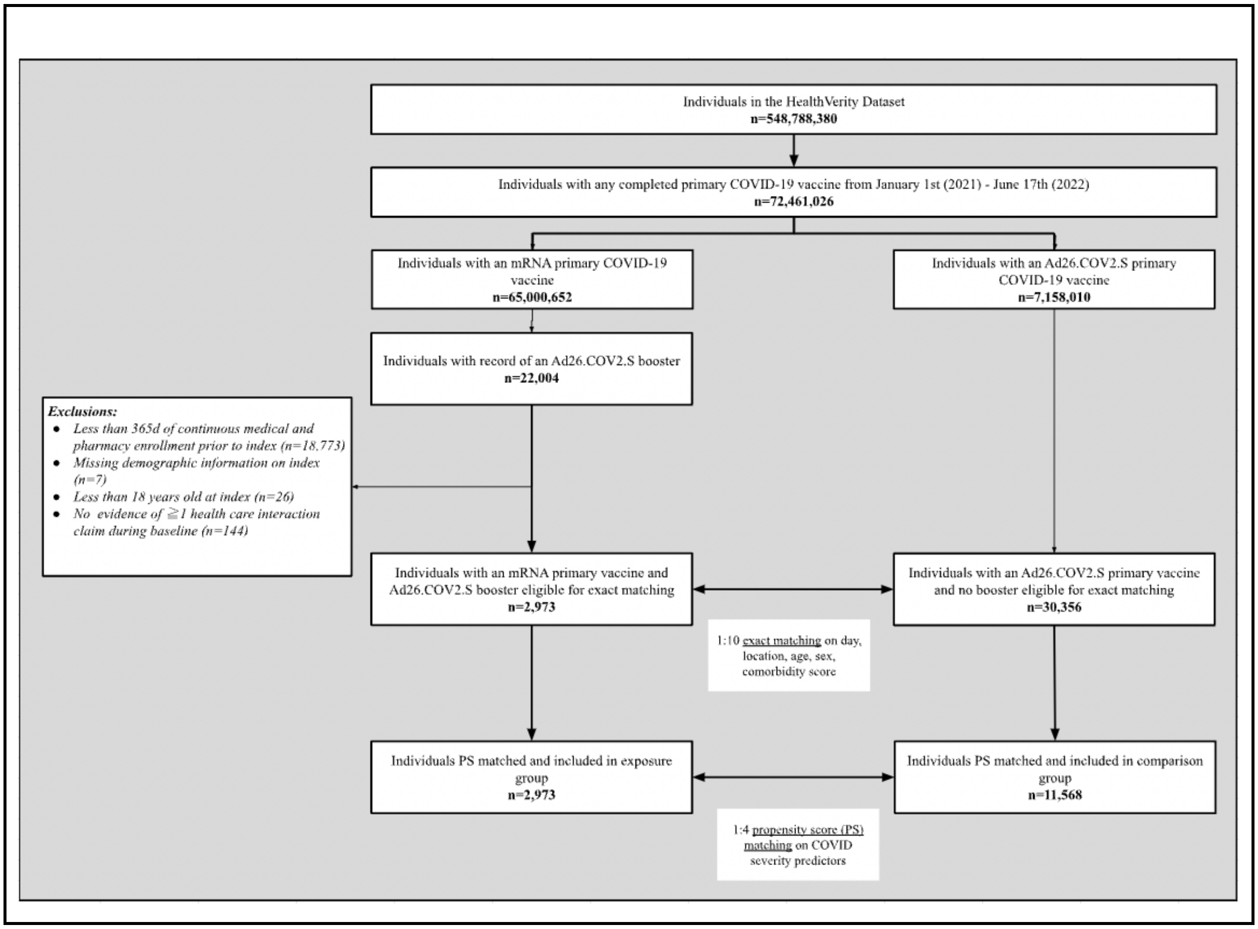

Figure S2. Study population flow diagram, mRNA + Ad26.COV2.S vs Ad26.COV2.S + no boost.

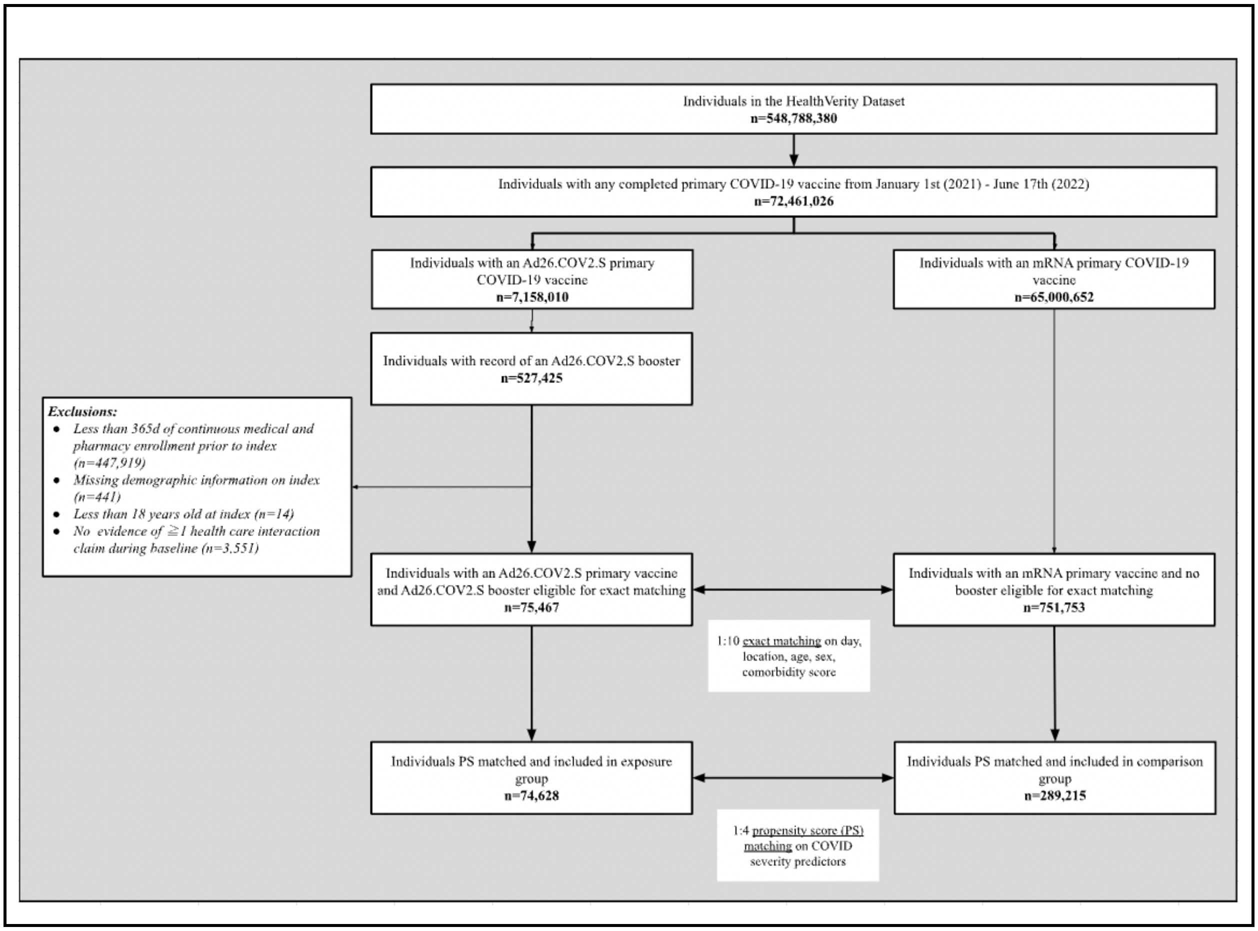

Figure S3. Study population flow diagram, Ad26.COV2.S + Ad26.COV2.S vs mRNA + no boost.

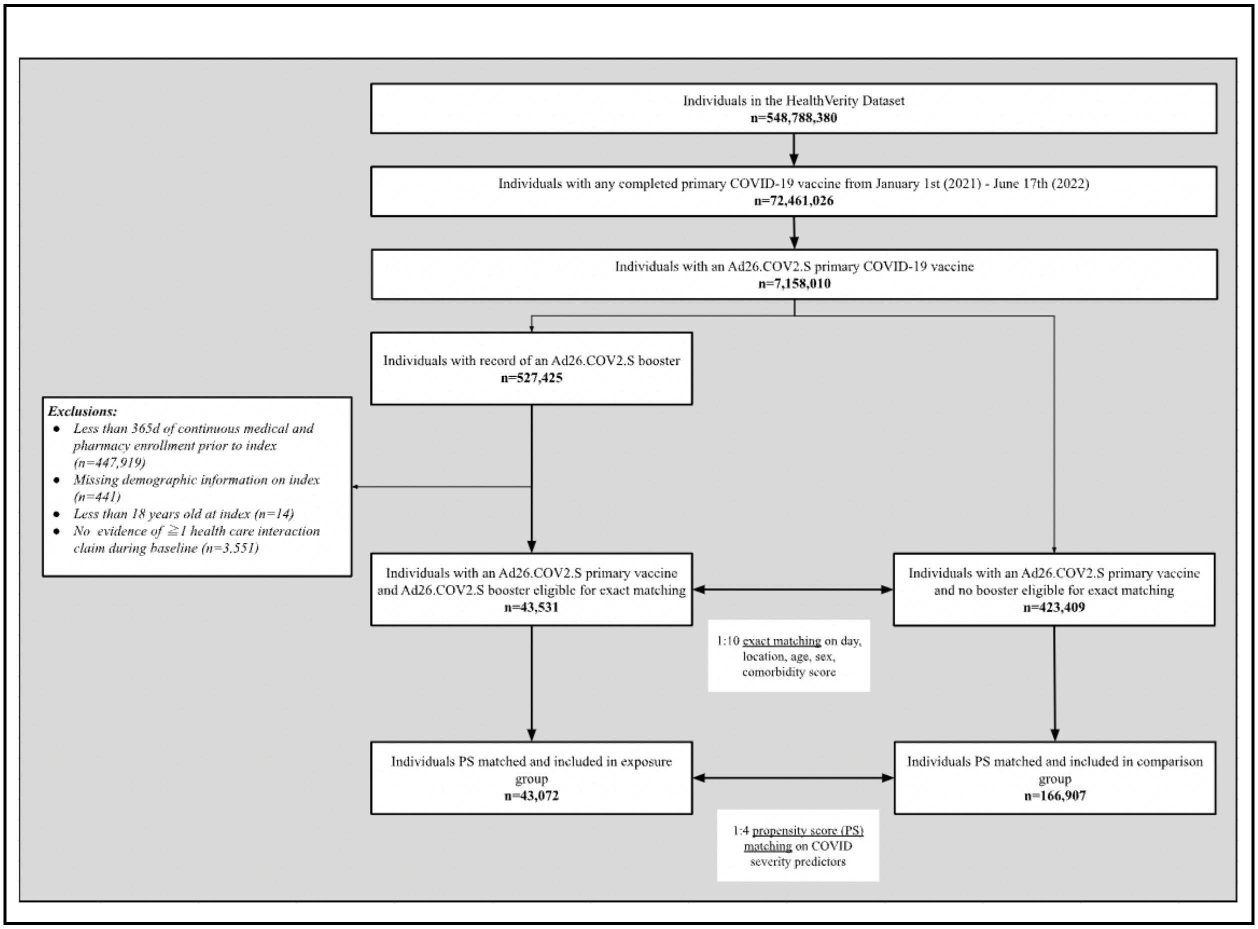

Figure S4. Study population flow diagram, Ad26.COV2.S + Ad26.COV2.S vs Ad26.COV2.S + no boost.
